# Lipoblastoma and Lipoblastomatosis of the Lower Leg

**DOI:** 10.1155/2014/582876

**Published:** 2014-09-15

**Authors:** Achmad Fauzi Kamal, I Gde Eka Wiratnaya, Errol Untung Hutagalung, Marcel Prasetyo, Evelina Kodrat, Wahyu Widodo, Zuhri Effendi, Kurniadi Husodo

**Affiliations:** ^1^Department of Orthopaedic and Traumatology, Cipto Mangunkusumo National Central Hospital and Faculty of Medicine, Universitas Indonesia, Jalan Diponegoro No. 71, Jakarta Pusat, Jakarta 10430, Indonesia; ^2^Department of Radiology, Cipto Mangunkusumo National Central Hospital and Faculty of Medicine, Universitas Indonesia, Jalan Diponegoro No. 71, Jakarta Pusat, Jakarta 10430, Indonesia; ^3^Department of Pathology, Faculty of Medicine, Universitas Indonesia, Jalan Salemba Raya No. 6, Jakarta Pusat, Jakarta 10430, Indonesia; ^4^Department of Orthopaedic and Traumatology, Gatot Soebroto Central Army Hospital, Jalan Abdul Rahman Saleh No. 24, Jakarta Pusat, Jakarta 10410, Indonesia

## Abstract

Lipoblastoma is a benign lesion of immature fat cells that is found almost exclusively in pediatric population. This tumor is a rare tumor that occurs in infancy and early childhood, accounting for less than 1% of all childhood neoplasm. It is more common in male than in female and often presents as an asymptomatic, rapidly enlarging, soft lobular mass on the extremity. Although benign, it gives great difficulty in its management, due to its extensions into different facial planes, especially in lipoblastomatosis. Thus, complete surgical excision is the treatment of choice.

## 1. Introduction

Lipoblastoma is a rare benign lesion of immature fat cells that is found almost exclusively in infancy and early childhood, accounting for less than 1% of all childhood neoplasms [1]. It commonly arises from extremity and trunk [2]. Lipoblastoma is more common in males than females and often presents as an asymptomatic, rapidly enlarging, soft lobular mass on the extremity [3, 4]. This paper reports two cases of lipoblastoma and one case of lipoblastomatosis of the lower leg. All cases were found in the childhood on the first three years of age.

## 2. Case Illustration

### 2.1. Case  1

One-and-a-half-year-old toddler presented with a lump in the right lower leg since the age of three months. At that time, her parents felt solid area on the right lower leg without pain. The mass started to look clear when she was ten months old. Subsequently, she was brought to the bonesetter, but there was no improvement. Two months before admission, she was brought to an orthopaedic surgeon in other province and then referred to our hospital.

**Figure 1:**
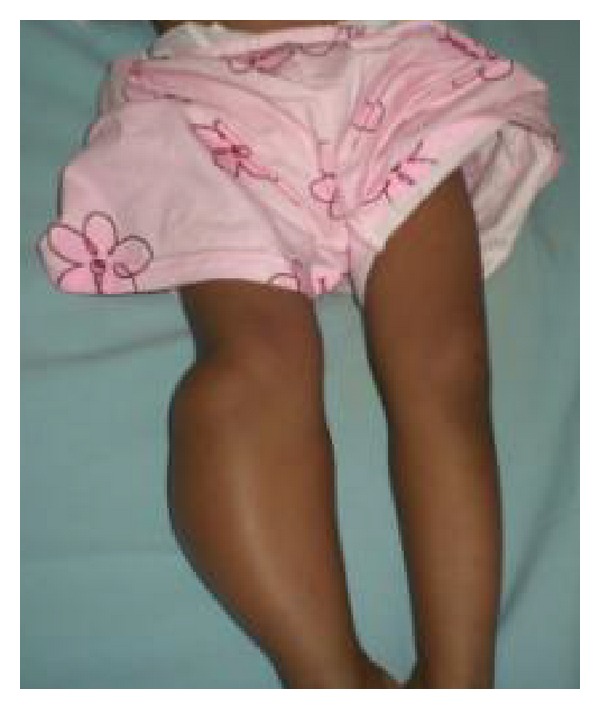
Clinical picture in Case  1 showed a big mass on the posterolateral side of right lower leg which had similar colour to the surrounding area.

On physical examination, there was a big mass on the posterolateral side of the right lower leg, with overlying skin colour similar to the surrounding area and no sign of inflammation. The tumour mass was smooth, painless, immobile, and sizing 12 × 5 × 3 cm (Figure 1). The knee movement was normal.

**Figure 2:**
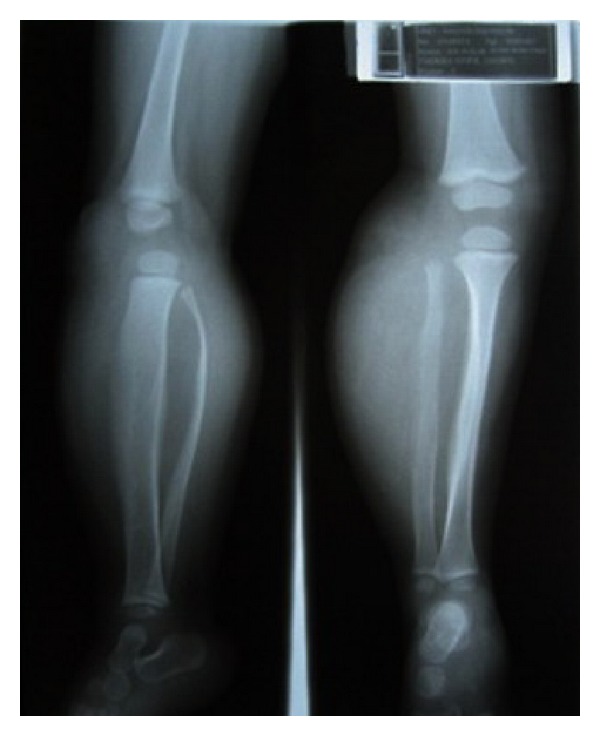
Plain radiograph of Case  1 showed a soft tissue mass. Both tibia and fibula were deformed, which gave an impression of a benign slow-growing lession.

**Figure 3:**
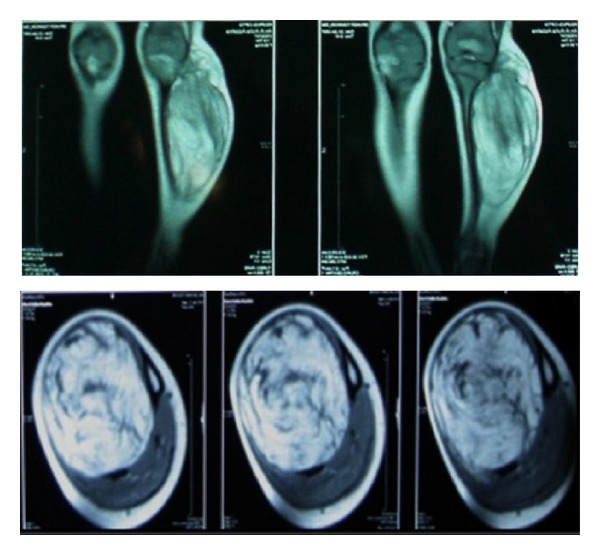
MRI of Case  1 in coronal and axial view of T1-weighted images. The soft tissue mass showed predominantly lipomatous tissue with some part of non-fatty component. The lession was well-circumscribed, with compression of adjacent bones and muscles.

Radiograph showed localized soft tissue mass with radiolucent component. Bowing deformity was noted on both tibia and fibula, along with non-aggressive periosteal thickening (Figure 2). Magnetic resonance imaging (MRI) on that area showed a well circumsribed intermuscular soft tissue mass, with predominant fatty component. The non-fatty component were consistent with myxoid tissue. Bone deformity was nicely depicted without any sign of destruction (Figure 3). Fine needle aspiration biopsy (FNAB) result was suspicious infantile fibromatosis. The final diagnosis after open biopsy was lipoblastoma.

**Figure 4:**
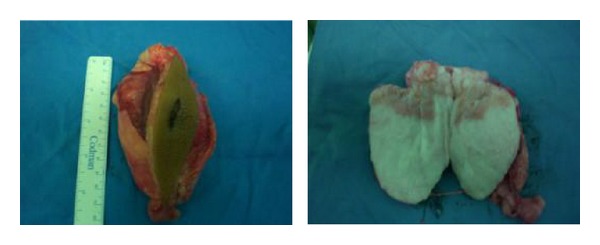
Macroscopic pictures of the tumour after surgical excision in Case  1.

**Figure 5:**
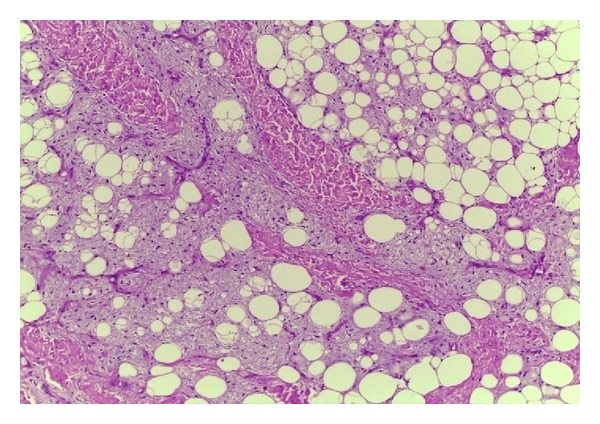
Histopathology of the specimen in Case  1. The tumour consists of mature adipose tissue and myxoid areas, with lacy network of small blood vessels (Hematoxylin and Eosin ×100).

The mass was completely excised through posterolateral approach; no adhesion to the surrounding tissue was found. The specimen consisted of a well-circumscribed, soft yellowish-white mass; the macroscopic features were suggestive of lipoblastoma (Figure 4). Microscopically, the tumor was lobulated and separated by connective tissue septa containing mature adipose tissue and myxoid mesenchymal tissue. The adipocytes were in various stages of differentiation, but there was no nuclear atypia. It was consistent with lipoblastoma (Figure 5).

### 2.2. Case  2

A two-and-a-half-year-old female toddler presented with a mass in her right lower leg since one year before. Initially, her parents noticed a pea sized mass growing bigger slowly. The onset was not associated with fever or trauma. The mass grew gradually until the size of a pumpkin on the lateral side of her right lower leg, which caused anxiety to her parents.

**Figure 6:**
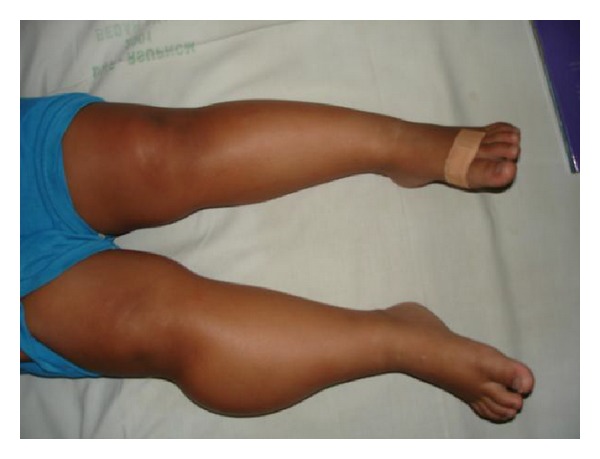
Clinical picture of the mass on lateral side of right lower leg in Case  2.

**Figure 7:**
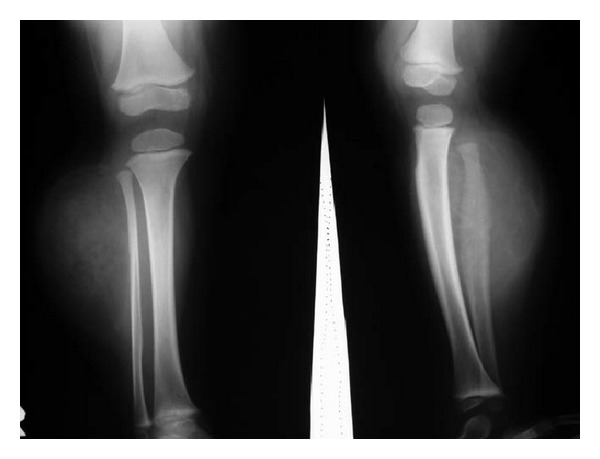
Plain radiograph of Case  2 showed a soft tissue mass with a more diffuse fatty-like radiolucent component. Although bowing deformity was not seen, periosteal thickening of the fibula was nicely depicted.

The mass was firm in consistency, with no tenderness, noncompressible, and nonpulsating. The overlying skin was glossy. Neurological examination distal from the mass was normal. Range of motion of right knee and ankle was normal (Figure 6). Radiograph of the right leg showed a soft tissue mass on the posterolateral side. Fibular bone was slightly deformed with periosteal thickening (Figure 7). Computerized tomography scan revealed that the soft tissue mass was suspected to be rhabdomyosarcoma. FNAB result was benign soft tissue lesion.

**Figure 8:**
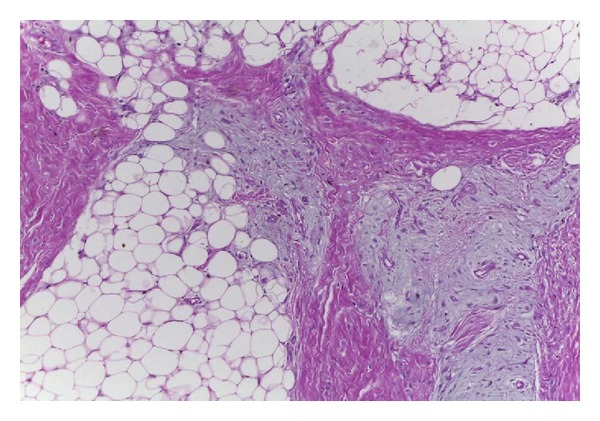
Histopathology of the specimen in Case  2. There was a lobulated mass separated by connective tissue septa consisting of mature adipose tissue, myxoid mesenchymal tissue, and lipoblasts. The myxoid area contains lacy network of small blood vessels (Hematoxylin and Eosin ×100).

Wide excision of the mass was performed similarly to the previous case. Gross pathology of the resected specimen showed an encapsulated, firm, lobulated mass with internal septations. On microscopic view, the tumour consisted of adipose tissue divided into lobules by fibrous septa, myxoid mesenchymal tissue, and lipoblasts. Immature myxoid cells were in the periphery and mature adipocytes in the center; the myxoid area contained lacy network of small blood vessels. The final diagnosis was lipoblastoma (Figure 8).

### 2.3. Case  3

**Figure 9:**
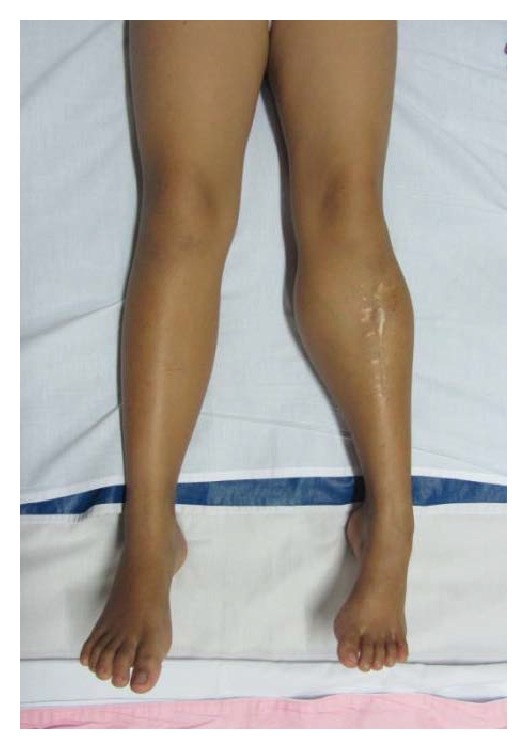
Clinical picture of the mass on lateral side of left lower leg in Case  3.

**Figure 10:**
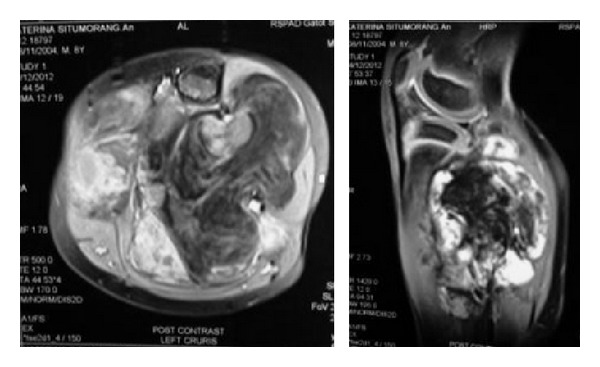
MRI of Case  3. Axial and sagittal view in T1-weighted images with fat saturation and contrast administration. The mass looked more infiltrative to surrounding muscles, with hypointense fatty component and mildly enhanced myxoid tissue.

An eight-year-old girl presented to our hospital with a painless mass below the left knee. Initially at the age of three years, her parents noticed that she had a mass on her left lower leg. She had undergone radiographic examination and excisional biopsy in other hospital. Several months after surgery, the mass recurred and became bigger. Two months before admission, she returned to the orthopedic surgeon who performed the surgery, and she was eventually referred to our hospital. The mass size was approximately 10 × 10 × 10 cm with smooth overlying skin, was nontender, and had ill-defined margin. There was scar from previous surgery and a fixed equinus deformity of the ankle (Figure 9). Radiographic examination showed a soft tissue mass in the proximal left lower leg. MRI showed a soft tissue mass with heterogenous signal intensity and ill-defined margin, which infiltrated the surrounding muscles without bone involvement (Figure 10).

**Figure 11:**
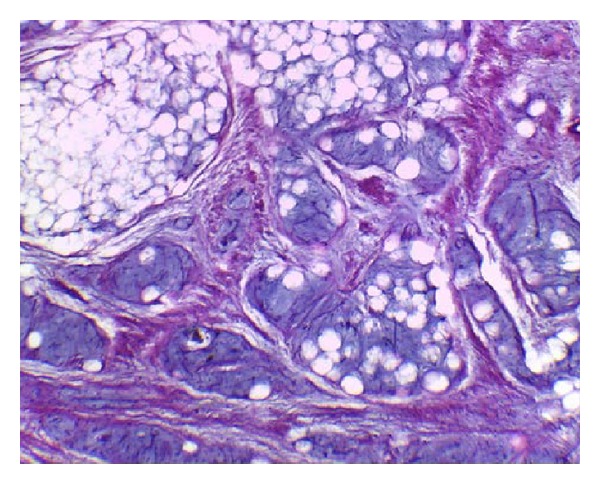
Histopathology of the specimen in Case  3. The tumor showed lobulated appearance consisted of mature adipocytes and myxoid parts (Hematoxylin and Eosin ×100).

The mass was excised through a posteromedial approach. There was adhesion of the mass to gastrocnemius and soleus muscles, tibialis posterior artery, and tibial nerve. Muscles were conserved except the part of the muscles which was attached to the mass. Microscopically the mass showed lobulated appearance consisted of mature adipocytes and myxoid parts (Figure 11). Based on clinical manifestations, result of MRI, and histopathology, we concluded it was a lipoblastomatosis.

## 3. Discussion

Lipoblastoma and lipoblastomatosis are rare benign tumors arising from fetal embryonal white fat [1]. The terms lipoblastoma and lipoblastomatosis were first used by Jaffè [5] in 1926 and Vellious [6] in 1958. Chung and Enzinger [2], in 1973, proposed the term lipoblastoma to be used for the circumscribed type. Another one, the infiltrative form, the term lipoblastomatosis was used for a diffuse multicentric type of this kind of neoplasm. The circumscribed type is the most common [7].

Lipoblastoma and lipoblastomatosis in our patients occurred in young age. These were consistent with the literature which said that the most common cases usually occur during the first 3 years of life with a median age of onset 1 year, and the oldest patient reported was 7 years of age [2]. Although it may also be found in newborn [8], the occurrence of lipoblastoma in patients older than 10 years of age is quite rare [2].

The tumors are commonly located in the extremities (30–70%) and the trunk (20–50%) [2]. Less common sites include head and neck area, mediastinum, mesentery, and retroperitonium. All tumors in our patients were located in the lower legs. These are similar to an article by Arda et al. (1993) [9]; they reviewed 84 cases reported in English literature and reported that the most common location of the tumors was in lower extremity (41%), followed by upper extremity (20%) and trunk (15%).

Lipoblastoma is commonly located in the subcutaneous or superficial soft tissue of the extremity and clinically may simulate lipoma [7, 10]. Lipoblastomas are frequently asymptomatic and painless, except when they impinge on surrounding structures, causing symptoms by their mass effect [3, 4]. Symptoms associated with these lesions are directly related to the location and size of the mass [10]. Although the tumors are benign, they often exhibit rapid growth [3, 4]. Our lipoblastoma patients (the first and second case) came to our hospital with a big mass whose location was deep in lower leg without any symptoms such as pain and difficulty in ambulation. Rapid growth of the mass encouraged their parents to seek medical attention. In the first case, the tumor compressed and caused thinning and bowing of the fibula. In fact, it grew more rapidly and more progressively than the one in the second case.

The diffuse type (lipoblastomatosis) originates in deep soft tissue and tends to infiltrate not only the subcutis but also the underlying muscle [10]. Therefore, it has a greater tendency to recur compared to lipoblastoma, particularly when the excision is incomplete [11]. It was the characteristic seen in Case  3 which had recurrence. The local recurrence in this patient occurred in less than 3 years after surgery. The mass arose again in the same area with a more solid consistency and with equinus deformity; these were taken into consideration for the diagnosis of lipoblastomatosis.

Magnetic resonance imaging is the best modality in evaluating benign fatty tumors in adults. Nevertheless, literature and reports of the MRI appearance of lipoblastoma in children are limited [4]. From the imaging point of view, lipoblastoma has variable appearances. These are most likely due to different stages of differentiation of lipoblastoma, which are attributable to the variable proportion of mature adipocytes within lobules and the amount of immature peripherally situated lipoblasts, fibrous septa, and myxoid tissues. The two most common appearances of lipoblastoma in MRI are a well-defined fatty mass and circumscribed soft tissue lesions embedded within large masses of fatty tissues [12].

It is very difficult to distinguish between myxoid liposarcoma and lipoblastomatosis based on radiology. Both have a predilection for lower extremities and an MRI image with heterogeneous signal intensity, because it contains little fat components and more nonlipoma structures. However, with the use of contrast, the image of liposarcoma will be homogeneous, whereas no or little contrast enhancement is shown in lipoblastoma. The age of onset also plays a role in distinguishing between those two. Lipoblastoma usually occurs at a younger age, while liposarcoma is found in older age. However, no age limit was found in both of them [13, 14].

Histopathology is the gold standard in establishing the final diagnosis [15]. Although FNAB could not determine the final diagnosis of soft tissue tumor, we performed it as an initial procedure to exclude malignancy. Final diagnosis should be confirmed by histopathology and should also be discussed with musculoskeletal radiologist and pathologist. Therefore, cases that were reported in our centre were always discussed in clinicopathological conference (CPC). On the first case, in CPC meeting, we decided to do open biopsy to confirm the diagnosis as lipoblastoma. In the second case, we also performed FNAB. Based on clinical symptom and sign, imaging, and previous experience of the first case, we performed similar surgical procedure. Arda and colleagues stated that biopsy should be considered to establish the preoperative diagnosis [9].

Although lipoblastoma is considered biologically benign, it may grow locally to an impressive size and lead to a significant mass effect. Therefore, complete surgical excision is the treatment of choice. Local recurrences are reported in 9% to 25% of cases and are usually associated with lipoblastomatosis cases which underwent incomplete excision [2, 6].

In conclusion, lipoblastoma is a poorly understood and uncommon soft tissue tumour in the infancy and early childhood. Although lipoblastoma is an uncommon childhood tumour, it should be taken into consideration as a differential diagnosis of tumor in the leg. Although it is benign, it gives great difficulty in its management, due to its extensions into different facial planes, especially in lipoblastomatosis. Thus, complete surgical excision is the treatment of choice.
